# The Timing, Factors, and Impact of USMLE Step 1 Becoming Pass/Fail on the Process of Choosing a Specialty

**DOI:** 10.7759/cureus.46844

**Published:** 2023-10-11

**Authors:** Jessica Hernandez-Moreno, Charissa Alo, Kian Habashi, Elli Tian, Edward Simanton

**Affiliations:** 1 Medical Education, Kirk Kerkorian School of Medicine at the University of Nevada Las Vegas, Las Vegas, USA; 2 University of Nevada Las Vegas, Office of Medical Education, Las Vegas, USA

**Keywords:** clinical clerkship, usmle step 1 pass/fail, medical education curriculum, pass/fail usmle step 1, medical specialty

## Abstract

Background

Choosing a medical specialty is an important decision. A combination of factors influenced this decision. Student characteristics and examination performances can influence this decision. With the transition of the United States Medical Licensing Examination (USMLE) Step 1 becoming pass/fail, it is important to analyze the specialty decision process.

Objective

The purpose of this multimethod study is to assess when in the curriculum students choose a specialty, what factors influence their decision, and the impact of USMLE Step 1 scores on a student’s assessment of competitiveness.

Methods

In February 2022, a survey was prepared and approved by the University of Nevada, Las Vegas (UNLV) Institutional Review Board (IRB). The survey contained multiple-choice questions and a free-response section. The survey was sent to the Class of 2022 and 2023 students at Kirk Kerkorian School of Medicine who follow a Longitudinal Integrated Clerkship. Descriptive statistics and one-sample t-tests were calculated.

Results

A total of 89 students completed the survey: 42 out of 60 students (70%) from the Class of 2022 and 47 out of 61 students (77%) from the Class of 2023. This study found that 78.8% of longitudinal interleaved clerkship (LInC) students committed to their specialty during the second half of the clinical year. The effects of positive and negative experiences during clerkships were most significantly different (p < 0.001).

Conclusion

The majority of LInC students arrive at their decision by the latter half of the clinical year. A variety of factors help students arrive at their decision. Our findings suggest that the pass/fail grading system will make it more difficult for students to assess their personal competitiveness.

## Introduction

Choosing a medical specialty is a complex decision that every medical student must make. A combination of student characteristics, values, priorities, clinical experiences, and curricula influence this decision [1,2]. Previous studies have shown varied results on when students make a decision and the factors influencing that choice [3-5]. The recent transition of USMLE Step 1 to a pass/fail grading system may add a layer of complexity to choosing a specialty. More research is needed on when medical students choose a specialty, what factors influence this decision, and how the new Step 1 pass/fail grading system will affect this process to further understand the current climate of choosing a medical specialty.

Current literature suggests that the majority of students reject the specialty intentions they declare when they begin medical school [3]. Even at the end of preclinical years, many students have not indicated a commitment to a certain specialty [3]. A recent study found that medical students are changing specialties in Year 4 (the last year) of medical school due to a shift in personal priorities [4]. Additionally, certain factors, including job satisfaction, lifestyle following training, and impact on the patient, have previously been ranked as highest at having an effect on specialty decisions [2].

Performance on board examinations can also heavily influence this process. Prior to matching into a specialty, a medical student must complete Step 1 and Step 2 of the United States Medical Licensing Examination (USMLE). Step 1 is an eight-hour multiple-choice exam that assesses whether a medical student understands and can apply important basic science concepts to the practice of medicine. Historically, Step 1 has been a graded assessment with a three-digit score intended only for licensure purposes [6]. However, this numeric score unintentionally evolved into a benchmark that allowed comparison and ranking of medical students and residency applicants [6].

Previous studies have reported that medical students use their USMLE Step 1 score to measure their level of competitiveness, pursue more competitive specialties, and revise their list of potential residency programs [7,8]. Not only was scoring well on these standardized exams associated with matching into more competitive specialties, but passing them on the first attempt was also correlated with a student’s competitiveness [8]. Altogether, it has been consistently shown that USMLE Step 1 scores have had a wide range of unintended consequences beyond the purpose of granting or denying licensure.

Driven by concerns for the well-being of medical students, the USMLE Step 1 grading system transitioned from a numeric score to pass/fail in February 2022 [9]. There has been widespread speculation about what this change could mean for medical students moving forward. Program directors have hypothesized that this transition may drive medical students to rely on subjective factors to assess their personal competitiveness [10]. Specifically, there may be more emphasis on funded research, first-author publications, and field-specific rotations, which are common among competitive applicants [11].

The purpose of this multimethod study is to assess when in the curriculum medical students choose a specialty, what factors influence their decision, and the impact of USMLE Step 1 scores on a student’s assessment of their competitiveness. The recent transition of USMLE Step 1 to a pass/fail grading system warrants further research on this dynamic process.

## Materials and methods

For this study, an electronic survey was created and distributed in accordance with an approved protocol by the University of Nevada, Las Vegas Institutional Review Board (IRB). The Office of Program Evaluation provided de-identified data from the survey including student feedback on their specialty selection and information about that process. The survey was sent to two cohorts at the Kirk Kerkorian School of Medicine at the University of Nevada Las Vegas (KSOM). At the time the survey was sent, the Class of 2022 had received their match results, and the Class of 2023 had completed the clinical curriculum (Phase 2) and were beginning the process of applying for residency through Electronic Residency Application Services (ERAS).

KSOM is a newly accredited medical school in the United States that accepted its inaugural class in 2017. The student body is highly diverse and is composed of a large percentage of first-generation and economically disadvantaged students. A table of demographics for the classes of 2022 and 2023 is provided below. At the time of this study, KSOM utilized the longitudinal interleaved clerkship (LInC) model rather than the traditional block rotation (BR) model during the third-year clinical phase.

Table 1 shows the demographics for the classes of 2022 and 2023.

**Table 1:** Demographics for the classes of 2022 and 2023

		2022	2023	Total
Ethnicity	Asian	23.7%	36.4%	30.4%
	Black	5.1%	4.5%	4.8%
	Hispanic	18.6%	12.1%	15.2%
	White	52.5%	47.0%	49.6%
Gender	Female	47.5%	51.5%	49.5%
	Male	52.5%	48.5%	50.5%
First Generation		25.4%	31.8%	28.8%
Economic Disadvantage		44.1%	37.9%	41.0%

Survey questions were designed to evaluate the following: When students finalized their choice of specialty; if students had previously made a specialty choice and then changed it; the importance of several factors and clerkship experiences on specialty decision, rated on a scale from 1 (not important) to 5 (very important); students’ perception of factors affecting competitiveness while applying to residency, rated on a scale from 1 (strongly disagree) to 5 (strongly agree); and a free-response section for additional comments.

Independent t-tests were calculated to determine if there was a difference in opinion between the two classes surveyed. For analysis of the survey, the questions were divided into three categorical groups. The three categorical groups included lifestyle, clerkship, and competitiveness. These groupings allowed us to calculate an average rating for each.

The survey used a Likert scale to rank student responses. Descriptive statistics were calculated for each scaled survey question, and the average rating for each group of questions was calculated. For example, “Lifestyle following training” was a question on the survey that students were to respond to. The answer choices for this question included “Not important”, “Less important”, “Somewhat important”, “Important”, and “Very important”. The lowest-ranking answer choice (i.e. “Not important”) received a value of 1 and the highest-ranking answer choice (i.e. “Very important”) received a value of 5. This particular question generated an average of 4.23. The group (i.e. Lifestyle Factors) average was 3.2. One-sample t-tests were calculated to explore the relationship between the rating of each survey question and the corresponding group average.

## Results

**Figure 1:**
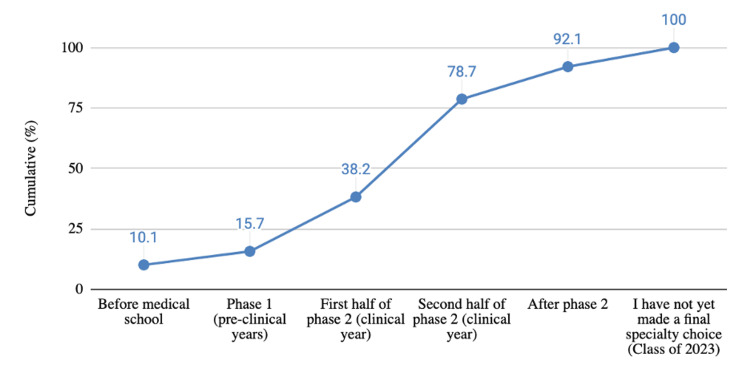
Cumulative percentage of KSOM students who finalized their specialty choice by respective points in the clinical curriculum KSOM: Kirk Kerkorian School of Medicine at the University of Nevada Las Vegas

A total of 89 students (N=89) completed the survey, 42 out of 60 students (70%) from the Class of 2022 and 47 out of 61 students (77%) from the Class of 2023. All of the 89 responses were used for this study. There was no statistically significant difference in the mean rating of questions or factors between classes, except for the question regarding the impact of specialty status/prestige on specialty choice. Given the lack of difference between classes, analysis was performed with both cohorts combined. We surveyed if in the LInC curriculum medical students commit to a specialty and the results are shown in Figure 1. A small portion (10.1%) of KSOM students indicated having known their specialty decision before medical school. By the end of the clinical year (clerkships), 78.7% of KSOM students indicated having chosen a specialty. Importantly, after the first half of the clinical year, there was the largest rise (40.5%) in specialty decisions being made. Another point in the curriculum that observed a large rise in specialty decisions occurred between Phase 1 and the first half of Phase 2, where 22.5% of students made their decision. Both these results point to the importance of exposure during the clinical years in the making of this decision.

**Figure 2:**
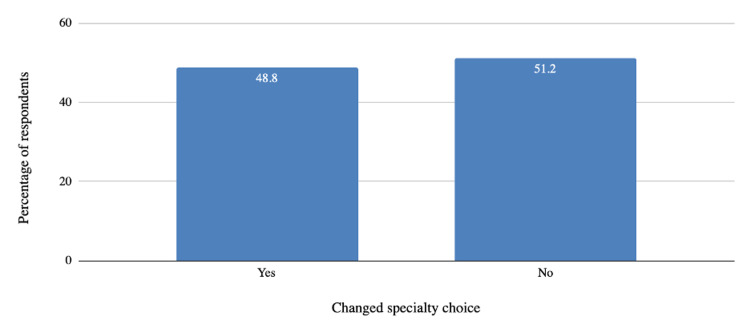
Percentage of KSOM students who changed their original specialty choice KSOM: Kirk Kerkorian School of Medicine at the University of Nevada Las Vegas

Additionally, we surveyed how many students changed their original specialty choice shown in Figure 2. Roughly half (48.8%) of the respondents had changed their original specialty choice.

**Figure 3:**
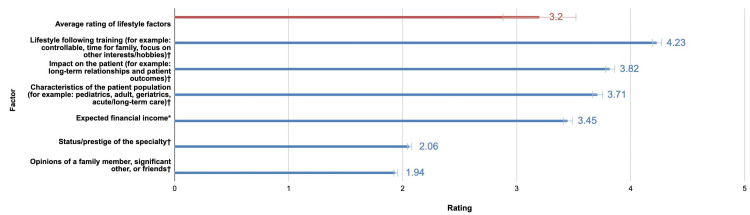
Mean and standard deviation of survey questions assessing the importance of lifestyle factors in specialty decision

We surveyed the importance of different lifestyle factors in specialty decisions shown in Figure 3. Lifestyle following training, impact on the patient, characteristics of the patient populations, and expected financial income were all rated more important than the mean of the lifestyle factors. Status/prestige of the specialty and opinions of a family member, significant other, or friends were rated lower than the mean of the lifestyle factors. All factors surveyed had differences from the mean of the lifestyle factors that were statistically significant (p < 0.05 for expected financial income; p < 0.001 for all other factors). Other factors affecting specialty choice that were mentioned in the free response section include long-term job or fellowship opportunities, length of training, ability to balance clinical responsibilities and research, residencies available in or near Las Vegas, and not matching into a preferred specialty. There was a statistically significant difference in the responses between the two cohorts regarding the impact of specialty status/prestige on specialty choice. The impact of specialty status/prestige on specialty choice (μ = 2.31 for c/o 2022, μ = 1.80 for c/o 2023; p = 0.015).

**Figure 4:**
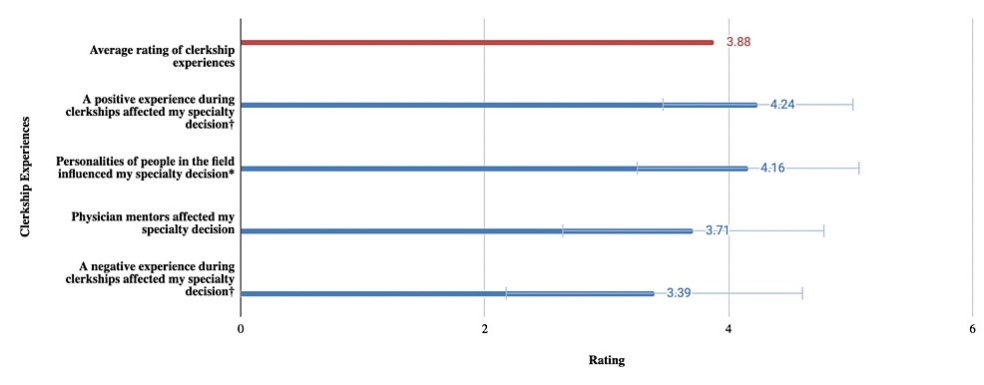
Mean and standard deviation of survey questions assessing the impact of clerkships on specialty decision

We surveyed the importance of different clerkship experiences shown in Figure 4. The effect of positive experiences during clerkships was most significantly different (p < 0.001) from the average rating of 3.88. The effect of the personalities of people in the field was also significantly different (p < 0.05). Other clerkship experiences affecting specialty choice that were mentioned in the free response section include satisfaction with the day-to-day workflow of a specialty and disliking the surgical environment.

**Figure 5:**
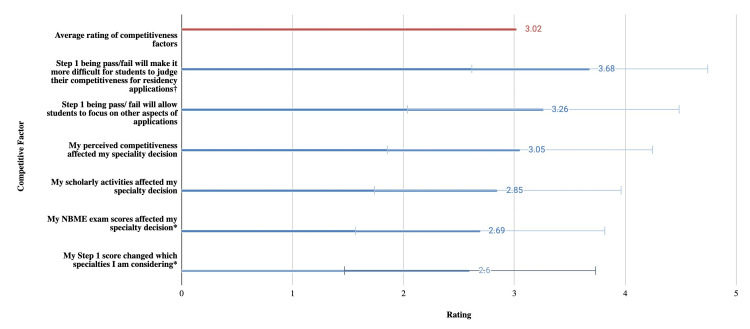
Mean and standard deviation of survey questions assessing the perception of factors affecting competitiveness

We surveyed different factors regarding competitiveness shown in Figure 5. The effect of Step 1 becoming pass/fail on students judging competitiveness was most significantly different (p < 0.001) from the average rating of 3.02. The effects of Step 1 and National Board of Medical Examiners (NBME) exam scores on assessing personal competitiveness (p < 0.05).

Lastly, the free-response section was intended to garner input on Step 1 becoming pass/fail. Within the free-response section, one student voiced “Time for self-care, wellness, exercise, family, travel” as a lifestyle factor that was important to them when choosing a specialty. Along these lines, another student noted “Ability to have a balance” as an important feat during their decision process.

In the free-response section, experiences during clerkships were brought to attention as one student noted “Getting along with colleagues” as important. Clerkship experiences were also acknowledged as a time to explore what the students wanted. One student mentioned, “When I finally tried the specialty I did not enjoy it as much as I thought I would.”

Another student noted, “It will make match more difficult coming from unknown schools like ours. Why would competitive residency programs take a risk on me when they have so little to gauge my competitiveness off of?” Another student stated, “Less certainty about what [students] [are] competitive for until much later in the year.”

Overall, the input on Step 1 becoming pass/fail reflected a combination of positive and negative comments, with the majority of comments expressing concerns. Specific concerns about the pass/fail grading system included: coming from a lesser-known school and having to wait until Step 2 to receive a numeric score. Only one comment reflected a positive outlook on this transition, stating that students may pursue a specialty based on genuine interest rather than being influenced by a score.

## Discussion

Timing

This study shows that a small percentage of LInC students choose a specialty before medical school (10.1%). There was an increase to 22.5% of students choosing a specialty after Phase 1 (pre-clinical years). There was a steady rise in students choosing a specialty in Phase 2, clinical years (Figure 1). Students in LInC, a teaching model used at KSOM, are simultaneously exposed to various specialties in two-week rotations. Figure 1 shows an increase of 78.8% of students choosing a specialty by the second half of the clinical year. There was a total of 40.5% of students making their decision between the first and second half of the clinical year. During this time, LInC students have been exposed to nearly every specialty in their training. This percentage is comparable but slightly lower than a study reporting roughly 85% of medical students indicating their specialty by the end of Year 3, clinical year [10]. This could indicate that LInC students take slightly longer to choose a specialty due to having to weigh and balance more factors earlier in their curriculum. This study is one of the first in current literature that explores the timing of when LInC students choose a specialty.

As shown in Figure 2, nearly half (48.8%) of subjects in this study changed their original specialty choice in the course of their curriculum. This is consistent with previous studies that show a majority of students are undecided about their specialty when they begin medical school and end up rejecting the specialty intentions they declared early in the curriculum [3].

Factors

Previous studies have examined factors related to choosing a specific specialty and have shown varied results [2,4]. Our study had the advantage of including 28.8% of first-generation medical students, which is well above the 12.4% national average reported by the Association of American Medical Colleges in 2022. Our childhood and upbringing greatly influence our ability to be oriented from an earlier age to lifestyle and career options. However, the diversity of our sample allowed us to survey a wide variety of factors that influence specialty choice. Figure 3 shows factors, including lifestyle following training, impact on the patient, patient characteristics, and financial income, rated as important. Whereas, factors including status/prestige of specialty and opinions of family and friends were considered less important. Students choosing family medicine ranked lifestyle as being the highest determining factor, whereas students who chose general surgery ranked job satisfaction and intellectual curiosity as most important [2,4]. This study explored factors considered important in general, not focusing on a specific specialty.

The opinion of a family member has been previously reported as a significant determinant in swaying a medical student into a specialty [12-14]. However, as Gill et al’s research implied, this factor was not ranked as the most statistically significant in comparison to other factors such as lifestyle and patient impact. Our findings concur with this and support the notion that choosing a specialty is a highly individualized process based on the student’s personal preferences. The self-reflection and self-assessment that occur throughout medical school allow students to see if their personality is better suited for certain specialties [12,15].

Figure 4 shows the impact of various clerkship experiences on specialty decisions. The effect of a positive experience and the personalities of people in the field were shown to be the most important factors. This supports previous research suggesting that a positive clerkship experience can have the effect of influencing a student’s decision [15]. Having a negative experience during clerkships seemed to be less important than the average rating. This differs from previous literature stating that negative stigmatizations and outlooks toward a specialty steer students away from it [14-16]. These findings suggest that a positive experience can help confirm a preference for a specialty, however, a bad experience does not necessarily drive a student away.

Step 1 becoming pass/fail

Figure 5 shows factors that affect a student’s perception of their competitiveness. Out of all factors, the effect of Step 1 becoming pass/fail was the most significant factor affecting a student's judgment of their competitiveness. The other significant factor identified as important was NBME exam scores.

Consequences of Step 1 moving to pass/fail grading mentioned in the free response section include a possible shift of emphasis toward a Step 2 score, more students taking research years for competitive specialties, and more weight given to medical school prestige. Possible uncertainty about personal competitiveness until later in the curriculum was also expressed. With step 1 becoming pass/fail for the class of 2025 and onward, it will be interesting to observe the effect of these changes and if they reflected the students’ expressions in this study.

The results of this study suggest that students have historically used Step 1 scores as a method to gauge their competitiveness and their likelihood of matching into a certain specialty. This supports previous studies also implying that Step 1 helped students narrow down their specialty interests [6-8].

## Conclusions

There have been recent changes to the grading system for the USMLE Step 1 exam, which has resulted in speculation about how this will impact medical students. The results of the current study suggest that Step 1 becoming pass/fail has made students feel uncertain about their ability to objectively measure their competitiveness. Additionally, this multimethod study showed that although KSOM uses an LInC training model, the majority of students will change their original specialty choice while making their final specialty decision during the second half of the clinical year when they have been exposed to all specialties in their curriculum. Furthermore, this study identified several factors that aid students in making a specialty decision including lifestyle following training, impact on the patient, and positive clerkship experiences.

Further research is necessary to explore the changing nature of arriving at a specialty decision as medical education continues to adapt and evolve. An emphasis on more qualitative research with cohorts from different medical schools can provide a more holistic picture of making a specialty decision when coming from a variety of backgrounds. This study contains several limitations. First, clerkship experiences vary across the country, and it is important to note that the survey results from this study do not speak for the experiences and viewpoints of medical students in general. Second, there are several different medical education models, KSOM is one of the few medical schools that offers the longitudinal interleaved clerkship (LInC) model for medical education. Third, although it is important to include a diverse population, this study surveyed students from one institution composed of a large percentage of first-generation and economically disadvantaged backgrounds, thus limiting the generalizability of these findings. Lastly, our results stem from the individualized experiences of only 89 students. Thus, interpretation is limited by the sample size of this study.
